# Formal Educational Preparation and Continuing Professional Development Needs in Specialized Palliative Care Nursing: A Nationwide, Cross-Sectional Study

**DOI:** 10.3390/nursrep16050175

**Published:** 2026-05-19

**Authors:** Tina Košanski, Marijana Neuberg, Mateja Križaj Grabant, Tomislav Meštrović

**Affiliations:** 1 University Centre Varaždin, University North, 42000 Varaždin, Croatia; ticikac@unin.hr (T.K.); mneuberg@unin.hr (M.N.); mkrizaj@unin.hr (M.K.G.); 2 Institute for Health Metrics and Evaluation, University of Washington, Seattle, WA 98105, USA

**Keywords:** palliative care, palliative care nursing, nursing education, continuing professional development, holistic care, nursing competence, cross-sectional study, Croatia

## Abstract

**Background:** Specialized palliative care requires nursing professionals to address the complex physical, psychological, social and spiritual needs of patients with advanced incurable illness. This study aimed to assess the perceived adequacy of formal educational preparation among nurses working in specialized palliative care services in the Republic of Croatia and examine its association with self-assessed knowledge and the perceived need for additional education. **Methods:** A nationwide cross-sectional survey was conducted among nursing professionals employed in specialized palliative care services across Croatia. Data were collected using a structured questionnaire assessing sociodemographic characteristics, perceived adequacy of formal education, self-assessed knowledge, as well as the need for additional education in physical, psychological, social and spiritual care domains. An Educational Sufficiency Discrepancy Index (ESDI) was calculated to quantify the difference between perceived educational sufficiency and continuing education needs. For inferential statistics significance was set at *p* < 0.05 (two-tailed). **Results:** Among the 194 nursing professionals who participated in the study, perceived educational sufficiency was highest in the physical domain (87.5%), where it exceeded the reported need for additional education (31.6%). Negative discrepancies were observed in social (−12.9) and spiritual care (−17.6), indicating perceived educational deficits. Representation of physical care content in formal education was significantly associated with higher self-assessed knowledge across several domains (physical *p* < 0.001; psychological *p* = 0.008; social *p* < 0.001; spiritual *p* = 0.008). No significant associations were found between self-assessed knowledge and age, work experience or level of education. **Conclusions:** Formal nursing education alone may not fully meet the multidimensional competency requirements of specialized palliative care practice. Strengthening structured continuing professional development, particularly in psychosocial and spiritual care, may support holistic palliative care delivery and sustained professional competence.

## 1. Introduction

Specialized palliative care represents one of the most demanding segments of the contemporary healthcare system, as it aims to integrate physical, psychological, social and spiritual dimensions of care for individuals with incurable, advanced illnesses [1]. According to the definition of the World Health Organization (WHO), palliative care aims to improve the quality of life of patients and their families through the prevention and relief of suffering, by means of early identification and optimal management of pain and other problems—physical, psychosocial and spiritual [2]. In this context, nursing professionals working in specialized palliative care services play an indispensable role, as they are continuously present at the patient’s side, responsible for nursing care while coordinating multidisciplinary interventions.

The competence of nursing professionals in palliative care has, therefore, been recognized as one of the fundamental determinants of overall care quality [3]. National and international guidelines emphasize the need for systematic education of nurses, as well as other professionals who have a role in palliative care—and this education should cover all domains of holistic care, including communication, symptom assessment, psychological support and spiritual care [4]. Nevertheless, research indicates considerable variability in the inclusion of palliative care content within formal nursing curricula, most notably in the domains of psychosocial and spiritual care [5,6,7,8,9].

In the Republic of Croatia, the course “Palliative Nursing Care” has been a mandatory component of the undergraduate nursing curriculum since 2014 [10]. Despite this, there is still a need for continuous professional development, especially considering that palliative care is an inherently dynamic and emotionally demanding field of practice. Also, the depth and practical implementation of these domains, particularly psychosocial and spiritual care, may vary across study programs [11,12,13]. The domains of psychological and spiritual care are contentious areas, where competencies are more difficult to operationalize and where professionals often express uncertainty despite having received formal education [11,12,13,14]. In addition to undergraduate education, nursing professionals may access continuing professional development on the topic through short courses, workshops and institutional training programs; still, these opportunities are not always systematically standardized or equally accessible across healthcare settings [10,11].

In Croatia, the development of specialized palliative care services has intensified over the past decade; however, systematic assessments of the alignment between formal educational preparation and the need for continuing professional development in this field remain limited. While previous analyses from the same dataset have examined specific dimensions of specialized palliative care practice, particularly spiritual care proficiency and practices [11], the present study addresses a distinct research question focused on the relationship between the perceived adequacy of formal educational preparation among nurses employed in specialized palliative care services in Croatia, their perceived need for additional education, as well as the association of these variables with self-assessed knowledge in the physical, psychological, social and spiritual domains of care. In addition, the study sought to operationalize the discrepancy between perceived educational sufficiency and continuing education needs.

## 2. Materials and Methods

### 2.1. Study Design

This study employed a quantitative cross-sectional design. It represents an analysis of data collected as part of a broader doctoral research project examining the implementation of holistic nursing care in specialized palliative care services in the Republic of Croatia. While a previous publication based on the same dataset focused on specific dimensions of specialized palliative care, the present analysis specifically examines perceived adequacy of formal education, continuing education needs, as well as their association with self-assessed knowledge across physical, psychological, social and spiritual domains.

### 2.2. Setting and Participants

The study was conducted in specialized palliative care services across the Republic of Croatia, including palliative care coordinators, members of mobile palliative care teams, nurses in inpatient units within health centers, hospice settings, and palliative care departments.

Because comprehensive records of nurses employed in specialized palliative care services were not available, all institutions providing palliative care beds at the secondary and tertiary levels, as well as healthcare centers across all Croatian counties, were contacted to obtain estimates of nursing staff. More specifically, healthcare institutions providing specialized palliative care services were identified based on publicly available data from the Croatian Health Insurance Fund, which listed 29 institutions with palliative care beds in the Republic of Croatia. All identified institutions were contacted via email to obtain information on the organization of palliative care services, including whether palliative care was provided within dedicated units or integrated into other departments. Estimates of nursing staff included nursing professionals serving as palliative care coordinators, members of mobile palliative care teams, and those working in inpatient units with palliative care beds at the primary healthcare level. Based on the information collected, it was estimated that 313 nurses with varying educational backgrounds were employed in specialized palliative care services in Croatia during the study period.

More specifically, according to available national data, palliative care at the primary healthcare level included 44 coordinators and 40 nurses in mobile palliative care teams; however, one mobile team was inactive during the study period and was excluded. In addition, two primary healthcare centers operated inpatient units with palliative care beds, employing a total of 53 nursing professionals. As no publicly available registry of nurses employed in specialized palliative care services exists, additional data on workforce size were obtained through direct communication with assistant directors for nursing across institutions. Based on these data, it was estimated that approximately 178 nurses were employed in specialized palliative care services at secondary and tertiary levels, and 135 at the primary level, yielding a total estimated population of 313 nurses. It should be noted that this represents a dynamic population subject to fluctuation due to workforce mobility and leave.

Of the 40 institutions providing specialized palliative care services across all levels of care, ethical approval for participation was obtained from 33 institutions (23 primary, 9 secondary and 1 tertiary), which determined the final number of institutions included in the study and, consequently, the number of questionnaires distributed. A total of 286 questionnaires were then consequently distributed to all these nurses that could have been reached, and 194 correctly completed questionnaires were returned (response rate 67.8%). Prior to the main study, a pilot study was conducted on a non-random sample of 22 participants (September–October 2022) to assess the clarity and reliability of the instrument.

Participants were eligible for inclusion if they were general care nurses holding a bachelor’s, master’s or doctoral degree in nursing and were actively employed in specialized palliative care services at the time of the survey. This included nursing professionals working as palliative care coordinators, members of mobile palliative care teams, nursing staff in inpatient clinics within health centers that provide palliative care beds, as well as nurses employed in palliative care units or hospices. Palliative care coordinators were eligible only if they were also directly involved in patient care through participation in mobile palliative care teams during the study period.

Questionnaires were distributed by post following prior coordination with designated institutional contacts. Data collection was conducted over a six-month period, from January to June 2023.

### 2.3. Measuring Instrument

Data was collected using a structured questionnaire developed for the doctoral study based on a review of the relevant scientific/professional literature and policy documents on holistic and palliative nursing care, with items designed to capture four core domains (physical, psychological, social, and spiritual). The instrument underwent expert review to ensure conceptual relevance, clarity and domain coverage. Previously validated components of the instrument primarily included subscales assessing the frequency and confidence of spiritual care practices [11], and items published for the first time here appraise perceived adequacy of formal education, self-assessed knowledge, and perceived need for additional education. Expert review was pursued to ensure conceptual relevance and clarity.

Consequently, the questionnaire comprised several sections. The first section collected sociodemographic and professional data, including age, gender, years of professional experience in healthcare and palliative care, level of education, work setting, and organizational model. The second section assessed the frequency of holistic nursing interventions across physical, psychological, social, and spiritual domains using a 5-point Likert scale (1—never to 5—always). In the third section, participants self-assessed their knowledge of interventions within each domain on a 5-point Likert scale (1—very poor to 5—excellent). Of note, self-assessed knowledge was operationalized as a subjective evaluation of competence within each domain, reflecting perceived rather than objectively measured knowledge.

The fourth section examined the perceived adequacy of formal education, asking participants to indicate whether they felt sufficiently educated during their formal nursing training to manage patients’ physical, psychological, social, and spiritual needs (1—strongly disagree to 5—strongly agree). The final section evaluated the perceived need for additional education, measuring the extent to which participants considered further training necessary to adequately address each dimension of holistic care, also using a 5-point Likert scale. These constructs were measured using single-item indicators for each domain, allowing direct comparison between perceived educational sufficiency and demand for additional training across domains of care.

Internal consistency of the subscales in the broader dataset demonstrated satisfactory reliability (Cronbach’s α ranging from 0.721 to 0.898 across domains), with previously published subscales (e.g., spiritual care frequency and confidence) showing good reliability (α = 0.777 and α = 0.832, respectively).

### 2.4. Ethical Considerations

Ethical approval for the overarching doctoral study was obtained from the relevant institutional ethics committee (approval number: FZV-282/2021, date of approval: 29 October 2021, start of data collection: 9 January 2023). Additional approvals were secured from healthcare institutions providing specialized palliative care services. Participation was voluntary and anonymous. Participation was voluntary and anonymous, and all participants received written information about the purpose of the study, confidentiality assurances, as well as the right to withdraw at any time without consequences. Written informed consent was obtained prior to participation.

### 2.5. Statistical Analysis

Data were analyzed using IBM SPSS Statistics for Windows, Version 29.0.1 (IBM Corp., Armonk, NY, USA). Descriptive statistics were used to summarize categorical variables (frequencies and percentages) and continuous variables (means, standard deviations, and 95% confidence intervals). Likert-scale variables were summarized using distributional frequencies across response categories.

Normality of distribution was assessed using the Kolmogorov–Smirnov test. Likert-scale variables were treated as continuous due to the distributional properties observed in the data, which is a common approach in health research. Pearson’s correlation coefficient (r) was used to assess associations between self-assessed knowledge and age, years of professional experience, level of education, representation of holistic care content in formal education, and perceived need for additional education.

To examine the alignment between perceived educational adequacy and continuing education needs, an Educational Sufficiency Discrepancy Index (ESDI) was calculated as the difference between the proportion of participants who agreed/strongly agreed that they received sufficient formal education and those who agreed/strongly agreed that additional education was needed within each domain.

A *p*-value of <0.05 (two-tailed) was considered statistically significant.

## 3. Results

Table 1 presents participants’ responses regarding the representation of physical, psychological, social, and spiritual care content in their formal nursing education, as well as their perceived need for additional education in these domains of holistic care.

The majority of participants (87.5%) either agreed or strongly agreed that they had been sufficiently taught during their formal education to manage patients’ physical needs. Nevertheless, 31.6% reported that they agreed or strongly agreed that additional education is required to adequately address patients’ physical needs, while a further 35.2% neither agreed nor disagreed with this statement.

Regarding psychological care, 63.2% of participants agreed or strongly agreed that they had received sufficient formal education to manage patients’ psychological needs. However, 59.1% simultaneously agreed or strongly agreed that they require additional education in order to provide adequate psychological care.

In the social domain, 43.1% of participants agreed or strongly agreed that formal education had sufficiently prepared them to address patients’ social needs. In contrast, 56.0% agreed or strongly agreed that additional education is necessary to ensure adequate management of social needs.

With respect to spiritual care, 31.1% of participants neither agreed nor disagreed that they had been sufficiently educated in this area. A total of 37.3% agreed or strongly agreed that their formal education was adequate, whereas 31.6% disagreed or strongly disagreed. Notably, 54.9% agreed or strongly agreed that additional education is needed to adequately address patients’ spiritual needs.

Although for social and spiritual care, lower levels of perceived educational adequacy are accompanied by persistently high demand for further education, for psychological care, high perceived educational adequacy coexists with a similarly high perceived need for additional training. This suggests that perceived educational adequacy does not necessarily eliminate the perceived need for continued professional development; rather, a measurable discrepancy exists between formal educational preparation and ongoing competence demands.

To further examine the alignment between perceived educational adequacy and continuing training needs, an Educational Sufficiency Discrepancy Index (ESDI) was calculated as the difference between the proportion of participants who agreed or strongly agreed that they had received sufficient formal education and the proportion who agreed or strongly agreed that they required additional education (Table 2). The rationale for using such an approach lies in its intuitive interpretability, allowing direct comparison between two complementary dimensions of perceived preparedness (i.e., sufficiency of prior education and demand for further training) with the use of a simple and transparent metric. The subtraction of one proportion from another was intended as a pragmatic way to quantify the direction and magnitude of this perceived gap at the population level, rather than to construct a formally validated psychometric index.

Thus, by using the ESDI, physical care demonstrated a strong positive discrepancy (+55.9), indicating a clear sufficiency zone in which perceived educational preparation substantially exceeded perceived need for further training. Psychological care showed only a minimal positive discrepancy (+4.1), suggesting that perceived adequacy and the need for additional education coexist within this domain. In contrast, social care yielded a moderate negative discrepancy (−12.9), reflecting that perceived need for additional training exceeds perceived educational sufficiency. The largest negative discrepancy was observed in spiritual care (−17.6), indicating the most pronounced educational deficit among all domains of holistic nursing care.

Table 3 shows the associations between self-assessed knowledge of physical, psychological, social, and spiritual care interventions and age, years of work experience in the healthcare system, level of education, representation of holistic care content in formal education, as well as the need for additional education among nurses working in specialized palliative care services. No statistically significant correlations were identified between self-assessed knowledge across all four domains and age, total years of work experience in the healthcare system, or level of education.

However, knowledge of interventions aimed at addressing patients’ physical needs showed statistically significant positive correlations with the representation of both physical and psychological care content in formal education. Conversely, there was a statistically significant negative correlation with the perceived need for additional education to adequately manage patients’ physical needs. Specifically, participants who reported having been sufficiently taught during formal education about managing physical needs perceived themselves as having significantly higher knowledge not only in physical care (*p* < 0.001), but also in psychological (*p* = 0.008), social (*p* < 0.001) and spiritual care (*p* = 0.008).

Similarly, participants who reported sufficient formal education regarding psychological care needs perceived significantly higher knowledge in physical (*p* = 0.002), psychological (*p* < 0.001), social (*p* < 0.001) and spiritual care (*p* < 0.001). Adequate formal education in social care was significantly associated with higher self-assessed knowledge in psychological (*p* = 0.001), social (*p* < 0.001) and spiritual domains (*p* < 0.001). Likewise, sufficient education in spiritual care was significantly associated with higher perceived knowledge in psychological (*p* < 0.001), social (*p* = 0.007) and spiritual care (*p* < 0.001).

Regarding continuing education needs, participants who reported requiring additional education to adequately manage patients’ physical needs demonstrated significantly lower self-assessed knowledge in physical care (*p* = 0.001). Furthermore, significantly lower self-assessed knowledge in spiritual care was observed among participants who indicated a need for additional education to manage psychological (*p* = 0.040), social (*p* = 0.019) and spiritual needs (*p* = 0.022).

## 4. Discussion

This nationwide study provides a systematic insight into the relationship between formal educational preparation and the need for continuing professional development among nurses working in specialized palliative care services in the Republic of Croatia. The main findings indicate a pronounced discrepancy between perceived educational sufficiency and the need for ongoing professional development, particularly in the psychological, social, and spiritual domains of care, confirming that formal education does not eliminate the need for further professional development in specialized palliative care, as also demonstrated in other studies [15,16,17]. Of course, the findings of this study should be interpreted in light of the fact that all key variables were based on self-reported perceptions, reflecting subjective evaluations of educational adequacy and knowledge rather than objectively measured competence.

The most pronounced educational sufficiency was observed in the physical domain, where the perceived adequacy of formal education substantially exceeded the need for additional training. This finding is consistent with the traditional focus of nursing curricula on the physical aspects of care [18,19]. At the same time, positive correlations between the representation of physical care content in formal education and self-assessed knowledge reaffirm the importance of a structured curriculum in the development of professional competence.

In contrast, in the psychological domain, an almost complete alignment was observed between the perceived adequacy of education and the need for additional training (a minimal positive discrepancy). This finding suggests that even when nursing professionals perceive their education as adequate, they still recognize the need for further professional development. Such a pattern may be interpreted in light of the complexity of the communicative and emotional demands of palliative care. Previous studies indicate that providing psychological support to terminally ill patients requires continuous reflection, supervision, and additional training, particularly in the domain of communication skills, given that communication is an integral part of nursing care and becomes particularly complex in working with palliative patients [20,21,22]. Communication in palliative care often involves discussions about difficult and emotionally demanding topics such as disease prognosis, suffering, fear of death, loss of functionality, as well as changes in quality of life. Such conversations call for a high level of empathy, sensitivity, the ability to recognize patients’ verbal and non-verbal cues, as well as the capacity to adapt communication styles to patients’ individual preferences, emotional states and cognitive levels [8,20,21,22].

The negative discrepancy observed in the social and especially the spiritual domains indicates a clear perception of an educational deficit. Spiritual care demonstrated the largest negative ESDI, which is consistent with international studies identifying spiritual care as one of the least represented areas in both formal nursing education and nursing practice [11,23,24,25]. Despite the WHO’s recommendations regarding the integration of spiritual care into educational curricula, its implementation in practice often remains insufficient or inconsistent [26].

An important finding of this study is the absence of associations between self-assessed knowledge and age, work experience and/or level of formal education. This result supports contemporary understandings of competence as a construct that does not depend solely on years of experience or academic qualifications, but rather on the quality and relevance of educational content, and further on opportunities for continuing professional development [27]. At the same time, strong positive associations between the representation of specific domains in formal education and self-assessed knowledge confirm the key role of initial education as a foundation for professional confidence.

The finding that the perceived need for additional education correlates with lower self-assessed knowledge further supports the validity of the measured constructs. However, the coexistence of high perceived adequacy of education and a high need for additional education in the psychological domain suggests that continuing professional development should not be viewed solely as a mechanism to compensate for deficiencies in formal education, but also as an essential component of ongoing knowledge and skill development in complex fields of care such as palliative care.

In a broader context, these findings definitely have implications for the development and planning of specialized training programs and structured forms of continuing education for nursing professionals. The systematic introduction of supervision and standardized programs in spiritual and psychosocial care could contribute to reducing the discrepancies identified in this study. For example, this could include the development of nationally standardized continuing education modules focused on communication skills, spiritual assessment and psychosocial support, combined with regular case-based supervision sessions within palliative care teams. Moreover, strengthening structured educational pathways and professional development opportunities may also contribute to improving the visibility and professional recognition of nursing roles, which remains an important issue in Croatia as highlighted by previous research on public perceptions and gender bias in the nursing profession [28].

Several limitations of this study should be acknowledged when interpreting the findings. Although the study included a large and geographically diverse sample of nursing professionals across multiple levels of care, the absence of a comprehensive national registry and the requirement for institutional approvals meant that not all eligible participants could be reached; hence, the “nationwide” nature of the study should be interpreted with caution. Also, while the response rate of 67.8% is acceptable for survey-based research, it still introduces the possibility of non-response bias. The cross-sectional design does not allow causal relationships to be established between formal educational preparation, perceived need for additional education, and self-assessed knowledge. Moreover, the study relied on self-reported data, including self-assessed knowledge and perceptions of educational adequacy, which may be influenced by subjective interpretation, response bias or social desirability bias. The constructs analyzed represented subjective perceptions, and notwithstanding their common use in health workforce research, their psychometric properties (especially in single-item form) are inherently limited. A further limitation is the potential for common-method variance, as both perceived educational adequacy and self-assessed knowledge were measured using self-reported items within the same instrument, which may partly reflect response style or general self-confidence rather than true underlying relationships. It also has to be noted that the ESDI developed in this study represents an exploratory, derived analytical approach designed to operationalize the gap between perceived educational sufficiency and the need for additional training, which means the index has not yet undergone external validation or testing in other settings—therefore, its interpretation should be considered preliminary. A potential limitation is also that Likert-scale variables were treated as continuous despite their ordinal nature, which may introduce measurement imprecision and affect the robustness of correlation estimates. Considering the exploratory nature of the study, we did not apply formal correction procedures, so marginal *p*-values should be interpreted with appropriate caution. Finally, the study was conducted within the healthcare and educational context of the Republic of Croatia, which may limit the generalizability of the findings to other countries with different educational systems, workforce structures, as well as models of palliative care organization.

Despite these limitations, the nationwide scope of the study and the inclusion of nursing professionals from diverse palliative care settings provide valuable insights into perceived educational preparedness and continuing professional development needs within specialized palliative care nursing. Of course, the ESDI should be seen as an exploratory, descriptive indicator of the gap between perceived educational sufficiency and need for additional training, rather than as a validated metric, and its applicability requires further methodological validation. This means that future research should also include longitudinal designs in order to assess the impact of specific educational interventions on competency development, as well as incorporate objective measurements of knowledge alongside self-assessment. It would also be advisable to examine organizational and cultural factors that may influence perceptions of educational sufficiency and professional development needs. Ultimately, competence in palliative care nursing should be viewed as a process sustained through continuous professional development.

## 5. Conclusions

Our findings suggest that nursing professionals perceive gaps between formal educational preparation regarding the provision of comprehensive and holistic care in specialized palliative care services. Although initial education provides an essential foundation for professional competence, notable perceived deficiencies remain between perceived educational preparation and the competencies required in clinical practice, particularly in the psychological, social and spiritual domains of care.

This work highlights the importance of embedding structured continuing professional development into national palliative care education and workforce policies. Systematic investment in lifelong learning and competency development for nursing professionals working in specialized palliative care should be considered a key component of quality assurance and the sustainable development of palliative care services.

## Figures and Tables

**Table 1:** Frequency distribution of participants’ opinions regarding the representation of physical, psychological, social and spiritual care content in formal nursing education and the need for additional education.

Statement	Strongly Disagree/N (%)	Disagree/N (%)	Neither Agree nor Disagree/N (%)	Agree/N (%)	Strongly Agree/N (%)
During my formal education, I was sufficiently taught to manage patients’ physical needs.	0 (0.00%)	5 (2.60%)	19 (9.80%)	67 (34.70%)	102 (52.80%)
During my formal education, I was sufficiently taught to manage patients’ psychological needs.	6 (3.10%)	19 (9.80%)	46 (23.80%)	71 (36.80%)	51 (26.40%)
During my formal education, I was sufficiently taught to manage patients’ social needs.	4 (2.10%)	33 (17.10%)	73 (37.80%)	58 (30.10%)	25 (13.00%)
During my formal education, I was sufficiently taught to manage patients’ spiritual needs.	18 (9.30%)	43 (22.30%)	60 (31.10%)	42 (21.80%)	30 (15.50%)
I need additional education to adequately manage patients’ physical needs.	26 (13.50%)	38 (19.70%)	68 (35.20%)	44 (22.80%)	17 (8.80%)
I need additional education to adequately manage patients’ psychological needs.	10 (5.20%)	18 (9.30%)	51 (26.40%)	89 (46.10%)	25 (13.00%)
I need additional education to adequately manage patients’ social needs.	9 (4.70%)	13 (6.70%)	63 (32.60%)	81 (42.00%)	27 (14.00%)
I need additional education to adequately manage patients’ spiritual needs.	12 (6.20%)	20 (10.40%)	55 (28.50%)	74 (38.30%)	32 (16.60%)

**Table 2:** Educational sufficiency gap by domain to calculate Educational Sufficiency Discrepancy Index (ESDI).

Dimension	Enough Education (%)	Need Additional Education (%)	Educational Sufficiency Discrepancy Index (%)
Physical	87.5	31.6	+55.9
Psychological	63.2	59.1	+4.1
Social	43.1	56.0	−12.9
Spiritual	37.3	54.9	−17.6

**Table 3:** Association between self-assessed knowledge of physical, psychological, social, and spiritual care interventions and age, length of work experience in the healthcare system, level of education, the representation of physical, psychological, social, and spiritual care content in formal education, and the need for additional education among nurses working in specialized palliative care services: Pearson’s correlation coefficient (r). A *p*-value of <0.05 (two-tailed) was considered statistically significant (of note: statistically significant associations with small effect sizes should not be overinterpreted and their practical relevance may be limited).

Variable	Physical Knowledge	Psychological Knowledge	Social Knowledge	Spiritual Knowledge
	r/*p*-value	r/*p*-value	r/*p*-value	r/*p*-value
Age	−0.070/0.189	−0.090/0.072	−0.101/0.055	−0.018/0.728
Years of work experience in healthcare system	−0.063/0.236	−0.073/0.144	−0.085/0.092	−0.028/0.697
Years of work experience in palliative care	−0.037/0.497	0.012/0.815	−0.070/0.180	0.055/0.294
Completed level of education	0.066/0.274	−0.003/0.961	0.132/0.052	0.062/0.280
During formal education I was sufficiently taught to manage patients’ physical needs	0.342/<0.001	0.153/0.008	0.231/<0.001	0.154/0.008
During formal education I was sufficiently taught to manage patients’ psychological needs	0.184/0.002	0.241/<0.001	0.253/<0.001	0.247/<0.001
During formal education I was sufficiently taught to manage patients’ social needs	0.070/0.237	0.186/0.001	0.198/<0.001	0.269/<0.001
During formal education I was sufficiently taught to manage patients’ spiritual needs	0.035/0.545	0.206/<0.001	0.148/0.007	0.297/<0.001
I need additional education to adequately manage patients’ physical needs	−0.199/0.001	−0.040/0.469	−0.094/0.090	−0.052/0.347
I need additional education to adequately manage patients’ psychological needs	−0.060/0.310	−0.078/0.162	−0.057/0.313	−0.115/0.040
I need additional education to adequately manage patients’ social needs	−0.027/0.645	−0.075/0.177	−0.059/0.295	−0.132/0.019
I need additional education to adequately manage patients’ spiritual needs	−0.034/0.566	−0.082/0.135	−0.045/0.421	−0.128/0.022

## Data Availability

The original data supporting the findings of this study are available from the corresponding author upon reasonable request.

## Public Involvement Statement

There was no public involvement in any aspect of this research.

## Guidelines and Standards Statement

This manuscript was drafted against the STROBE (Strengthening the Reporting of Observational Studies in Epidemiology) statement as the primary reporting guideline for cross-sectional research approach (source: [29]).

## Abbreviations

The following abbreviations are used in this manuscript:

ESDI	Educational Sufficiency Discrepancy Index
IBM	International Business Machines Corporation
WHO	World Health Organization
